# Novel Compound Heterozygous Variants of the *ABCC8* Gene Warrant Identification of Pancreatic Histology in Infant with Diazoxide-unresponsive Congenital Hyperinsulinism

**DOI:** 10.3390/children8100836

**Published:** 2021-09-23

**Authors:** Rana Al Balwi, Dalal Bubshait, Raed Al Nefily, Omar Al Ghamdi

**Affiliations:** Department of Pediatrics, King Fahad Hospital of the University, Imam Abdulrahman Bin Faisal University, Damam 31441, Saudi Arabia; dkBubshait@iau.edu.sa (D.B.); rmalnefeily@iau.edu.sa (R.A.N.); Omar.a.gham@gmail.com (O.A.G.)

**Keywords:** congenital hyperinsulinism, diazoxide, *ABCC8*

## Abstract

Congenital hyperinsulinism (CHI) is characterized by dysregulated insulin secretion, resulting in severe hypoglycemia. Mutations in the *ABCC8* and *KCNJ11* genes encoding KATP channels in beta cells of the pancreas are common among patients with CHI. Autosomal recessive CHI with diffuse involvement is the most common type of CHI among Saudi patients. It is relatively common for patients with autosomal recessive CHI to be medically unresponsive and undergo pancreatectomy. In this case report, we describe novel compound heterozygous variants in the *ABCC8* gene in a Saudi infant that caused diazoxide-unresponsive CHI. The variants included a monoallelic paternally inherited variant that has been previously reported to cause a focal form of CHI and a maternally inherited variant of unknown significance (VUS). The severity of CHI in this patient was mild over the one-year follow-up period, with a near-optimal glycemic response on a low dose of octreotide. We suspected an atypical subtype of histological involvement in the patient. In this report, we highlight the phenotypic spectrum of novel compound heterozygous variants in a patient with CHI and consider that the report can help establish the pathogenicity of the VUS.

## 1. Introduction

Congenital hyperinsulinism (CHI) is one of the most common causes of recurrent hypoglycemia in early infancy. CHI comprises a group of different genetic disorders arising from inappropriate insulin secretion. *KCNJ11* and *ABCC8* are key genes encoding the potassium ATP channel (KATP), which is involved in regulating insulin secretion from pancreatic beta cells. Diazoxide, a KATP channel opener, is the first drug of choice [1]. Three discrete defects in the genes are known to cause CHI. First, an autosomal recessive inheritance of two gene mutations leading to diffuse histologic changes in the pancreatic islets. This inheritance causes a severe neonatal onset of disease that is unresponsive to diazoxide, and these mutations are associated with diffuse histologic changes in the pancreatic islets [2]. Second, a single recessive mutation on the paternal chromosome resulting in a clone of beta cells, which may lead to loss of the normal maternal allele. This results in homozygosity for the recessive mutations [3] and the “two-hit defect” induces adenomatosis of the pancreas. Third, a rare autosomal dominant mutation usually causing a milder form of KATP hyperinsulinism that often presents after infancy and is responsive to diazoxide. There have been reports of dominant mutations in the KATP-encoding genes that lead to a severe, diazoxide-unresponsive disease [4,5]. The development of 18-F-PET/CT imaging has aided in elimination of the need for previously required preoperative diagnostic invasive surgical procedures. 18-F-PET/CT imaging helps distinguish between diffuse and focal CHI, as well as localize focal lesions. Unfortunately, the sensitivity, specificity, and positive predictive value of the 18-F-PET/CT method are not 100% [6], and pancreatic biopsy is the definitive diagnostic method to confirm focal involvement. The incidence of persistent CHI, reported to be one case per 50,000 live births, varies dependent upon the gene frequency of both mutant alleles and population consanguinity [7,8]. In a large cohort study [8] involving more than 500 patients referred for genetic testing of CHI in the UK, consanguinity was reported in 24 patients, and the pick-up rate of mutations within these patients was high (16/24, 67%). This is contrary to the higher incidence rates of CHI in consanguineous populations in Saudi Arabia (1 in 2675) and Kuwait (1 in 19,960) [9,10]. Notably, the diffuse form of CHI is the most common type of mutation inheritance in the Saudi population [11] and it is not uncommon for patients with homozygous or compound heterozygous autosomal-recessive CHI to have a severe presentation and failed medical treatment and eventually undergo pancreatectomy. Herein, we report an infant who had novel compound heterozygous variants in the *ABCC8* gene to highlight the phenotypic spectrum of these two variants in a patient with CHI.

A one-year-old girl was referred to our hospital at the age of 15 days because of frequent hypoglycemia since birth. She was born at term with a birth weight of 4.1 kg to a nonconsanguineous married couple and a primigravida mother who was not known to have any medical illness. At presentation, the patient weighed 3.8 kg (50th %), had a length of 62 cm (75th %), and had a head circumference of 41 cm (75th %). No dysmorphic features were revealed upon examination, she had a normal primitive reflex for her age, her heart examination results were normal, her abdomen was soft and non-distended with no palpable organs, and she had normal female genitalia. She was breast fed (30 mL) every 2 h with dextrose infusion of D15 w% 23 mL/h and glucose infusion rate (GIR) of 14 mg/kg/min. During the initial evaluation, she developed frequent episodes of hypoglycemia (<50 mg/dL); when a critical sample was obtained at a glucose level of 42 mg/dL, her blood glucose level 20 min after 30 mcg/kg-glucagon injection was 101 mg/dL, and her urine was negative for ketones. The results of the critical sample revealed an insulin level of 21.5 uU/mL, C-peptide of 3 ng/mL, ACTH of 213.1 pg/mL, and cortisol of 16.6 µg/dL. Growth hormone, free fatty acid, and blood ketone levels were not tested because the reagents were not available, and arterial blood gas showed no acidosis. The second confirmed critical sample at a glucose level of 47 mg/dL showed an insulin level of 11 uU/mL and a C-peptide level of 2 ng/mL. The dextrose concentration was upgraded to D18 w% 30 mL/h. Therefore, the GIR increased to 21 mg/kg /min plus oral feeding of 60 mL breast milk, with 2.5 g of Polycose in every 30 mL of breast milk. Diazoxide (10 mg/kg/day) with hydrochlorothiazide (2 mg/kg/day) was administered, and the patient continuously developed frequent low blood glucose levels below 70 mg/dL despite increasing the diazoxide doses up to 20 mg/kg/day for seven days without dextrose support. Because there was no response to diazoxide, octreotide (7 µg/kg/day) was administered every 6 h. The patient was transferred to a tertiary hospital for pancreatic PET scans where 68Ga-DOTANOC PET/CT scans were performed; however, the results were inconclusive. As the patient started to show a better glycemic response with octreotide, doses were gradually adjusted and increased to 11 mcg/kg/day every 6 h, and diazoxide administration was stopped. The patient started to have a good glycemic response with no hypoglycemia and reached full feed (100 mL) of milk formula and breast feeding plus carb CH (oligosaccharide formula) with every feed (GIR = 10 mg/kg/min). At the age of 44 days and before discharge from our hospital, a safety fasting test was performed. She had a fast tolerance for a 6 h duration where blood glucose was monitored every 20 min. The lowest glucose level during fasting was 58 mg/dL without symptoms, and other glucose levels ranged between 77 and 95 mg/dL. Parents refused gastrostomy and hired a nurse at home for overnight feeding and frequent blood glucose monitoring. Next-generation sequencing at the Fulgent Genetics laboratory (Table 1) revealed compound heterozygous variants in the *ABCC8* gene. Testing the unaffected parents confirmed compound heterozygosity, and targeted sequencing was performed on both DNA strands of the *ABCC8* gene. The reference sequence was as follows: *ABCC8*: NM_001287174.1 CENTOGENE laboratory. Based on the results of the serial blood glucose levels at home over a one-year follow-up, the patient rarely had an episode of low glucose level <70 mg/dL, despite infrequent hypoglycemia <50 mg/dL during early infancy due to infrequent post-feed vomiting. Hypoglycemia immediately resolved after feeding. Home blood glucose monitoring showed no spontaneous hypoglycemia as the patient was receiving a regular milk formula plus carb CH (oligosaccharide formula) every 3 h in the first three months of life. Only early morning hypoglycemia was observed, which reached 55 mg/dL, and was not associated with symptoms. Blood glucose levels were maintained in the range of 90–200 mg/dL (as per the father’s documentation in a log book). Once the patient started to have repeatedly observed blood glucose levels above 200 mg/dL, continuous overnight feeding was discontinued. In addition, the GIR decreased to 8 mg/kg/min. The octreotide dose was further adjusted and decreased to 7 µg/kg/day. Currently, the patient rarely experiences hypoglycemia overnight or in the early morning. However, an episode of asymptomatic hypoglycemia reached 62 mg/dL during the daytime after physical activity, as per the mother’s observation, and this occurred approximately twice over a two-month period. Therefore, daytime octreotide doses were increased by 25%. Furthermore, the patient’s current glucose requirement (GIR) is 8 mg/kg/min, and starch and oat have been introduced to her meals to ensure adequate glucose supplementation during daytime activity.

## 2. Discussion

This report demonstrates new compound heterozygous variants that caused diazoxide unresponsiveness in an infant who could respond to relatively low doses of octreotide. The paternally inherited mutation is known to be pathogenic and has been previously reported with 11p15 chromosomal deletion in a compound heterozygous state, resulting in the focal form of CHI [12]. The second mutation was a maternally inherited VUS; however, whether it acts recessively is currently not known. The paternally inherited mutation of the *ABCC8* gene in cases of CHI can be either biallelic or monoallelic [13,14,15]. Biallelic *ABCC8* mutations result in the diffuse form of CHI, whereas monoallelic mutations can result in either asymptomatic conditions (when recessive) or focal forms of CHI (if paternally inherited and associated with a loss of the somatic maternal 11p allele). Alternatively, monoallelic mutations can lead to the diffuse form of CHI if it is dominant. Diazoxide is usually ineffective in treating patients with CHI due to biallelic recessive mutations in the genes encoding KATP channel or presence of monoallelic focal form of disease [13,14]. In our case, the parents were asymptomatic, and there were no other affected members in the family. Therefore, we speculated that these two inherited variants could act recessively, leading to diffuse CHI or an atypical subtype of histological involvement, as predicted by the mild clinical presentation and diazoxide unresponsiveness. Although diffuse CHI due to biallelic mutations in KATP-encoding genes typically presents with a severe phenotype, milder phenotypes for both compound heterozygous and homozygous cases have been reported previously [16], wherein patients were managed medically and did not require pancreatomy. Hence, it was concluded that the maternal mutation is recessive and that this case is an example of a biallelic, compound heterozygous mutation of the KATP channel presenting with a mild phenotype. Alternatively, the maternal VUS may be benign, and there is presumably a postzygotic loss of heterozygosity within the pancreas that results in the focal form of CHI. High-resolution 18-F-DOPA PET/CT is considered the standard of care for investigating focal lesions; however, this imaging technique was not available when the patient was admitted at the hospital and therefore, 68Ga-DOTANOC PET/CT was performed instead, which gave inconclusive results. To the best of our knowledge, focal hyperinsulinism has not been previously reported in the Saudi population and, given the high likelihood of a cure, a definitive confirmation test to identify the actual pancreatic pathology is essential. Consequently, as pancreatic biopsy is highly recommended in focal CHI, we initiated the transfer of the patient to a specialized center for CHI; however, the process was later declined by the family. Arya et al. reported that mutations in *ABCC8/KCNJ11* can manifest as a wide spectrum of CHI with focal involvement in 45% of patients. However, the heterogeneous clinical picture could be due to the modification of genes or other unknown factors [17]. Furthermore, previously reported cases of CHI with maternally inherited *ABCC8* VUS with a known paternal pathogenic recessive mutation have led to pitfalls and challenges associated with diagnosis, medical management, and have raised the question of whether imaging studies are warranted in such cases [18]. Novel compound heterozygous variants in the *ABCC8* gene could present with variable severity. The purpose of reporting this case is to (1) help establish the pathogenicity of the novel VUS, (2) demonstrate the spectrum of severity of possible compound heterozygous *ABCC8* mutations, and (3) describe the possibility of postzygotic loss of heterozygosity that leads to pancreatic focal lesions or a typical subtype, which has not previously seen in CHI cases within the Saudi population. However, the possibility of this maternal VUS being pathological exists, as the autosomal recessive mode of inheritance, which leads to diffuse HI, is considered the most common cause of CHI among this ethnic group. In summary, we recommend obtaining a definitive diagnosis of CHI through pancreatic biopsy for determining the best medical and surgical management.

## Figures and Tables

**Table 1:** Results of genetic analysis (*ABCC8* mutations).

Variant	Inheritance	Zygosity	Interpretation	Reference Gene
c.4238C>T p.Pro1413Leu	Paternal	Heterozygous	Likely pathogenic	NM_000352.4
c.2514T>A p.Ser838Arg	Maternal	Heterozygous	Unknown significance (VUS)	NM_000352.4

VUS: variant of unknown significance.
